# The Biomechanical Study of Extraforaminal Lumbar Interbody Fusion: A Three-Dimensional Finite-Element Analysis

**DOI:** 10.1155/2017/9365068

**Published:** 2017-09-26

**Authors:** Mingjie Yang, Guixin Sun, Song Guo, Cheng Zeng, Meijun Yan, Yingchao Han, Dongdong Xia, Jingjie Zhang, Xinhua Li, Yang Xiang, Jie Pan, Lijun Li, Jun Tan

**Affiliations:** ^1^Department of Spine, Shanghai East Hospital, Tongji University School of Medicine, No. 150 Jimo Road, Shanghai 200120, China; ^2^Department of Traumatology, Shanghai East Hospital, Tongji University School of Medicine, No. 150 Jimo Road, Shanghai 200120, China

## Abstract

**Objective:**

Finite-element method was used to evaluate biomechanics stability of extraforaminal lumbar interbody fusion (ELIF) under different internal fixation.

**Methods:**

The L3–L5 level finite-element model was established to simulate decompression and internal fixation at L4-L5 segment. The intact finite model was treated in accordance with the different internal fixation. The treatment groups were exerted 400 N load and 6 N·m additional force from motion to calculate the angular displacement of L4-L5.

**Results:**

The ROMs were smaller in all internal fixation groups than those in the intact model. Furthermore, the ROMs were smaller in ELIF + UPS group than in TLIF + UPS group under all operating conditions, especially left lateral flexion and right rotation. The ROMs were higher in ELIF + UPS group than in TLIF + BPS group. The ROMs of ELIF + UPS + TLFS group were much smaller than those in ELIF + UPS group, and as compared with TLIF + BPS group, there was no significant difference in the range of experimental loading.

**Discussion:**

The biomechanical stability of ELIF with unilateral pedicle screw fixation is superior to that of TLIF with unilateral pedicle screw fixation but lower than that of TLIF with bilateral pedicle screws fixation. The stability of ELIF with unilateral fixation can be further improved by supplementing a translaminar facet screw.

## 1. Introduction

Lumbar degenerative disease has been the main cause of lower back pain and leg pain in adults [1–3]. There are a large number of treatment options available including both conservative and operative approaches. A systematic review by Phillips et al. [4] indicates that lumbar spinal fusion can be an effective treatment strategy for patients who are refractory to conservative treatment. The clinical consensus of lumbar interbody fusion is that as far as possible, the posterior tensile structures should be retained and unnecessary trauma should be reduced to ensure postoperative short-term stability and long-term fusion rate [5, 6]. Therefore, transforaminal lumbar interbody fusion (TLIF) with bilateral pedicle screw (BPS) fixation has been considered as the classical surgical approach in the recent years. However, TLIF still needs to resect the inferior facet joint firstly to provide access for the resection of the superior facet joint to decompress the nerve root. We wonder if it is feasible to release the compressed nerve root by the direct resection of the superior facet joint with retaining the inferior facet joint. Hence, we deeply study the anatomical structure of the intervertebral foramen. Intervertebral foramen is a circular area formed by the semicircular notches between the two pedicles of vertically adjacent vertebral bodies. The anterior wall of the foramen is the intervertebral disc, the superior and inferior walls are the superior and inferior pedicle notches, respectively, and the posterior wall is the facet joint and joint capsule formed by the superior and inferior facet joints of adjacent vertebral bodies. The upper edge and ventral side of the superior facet joint are in close contact with the nerve root, which is an important anatomic factor leading to nerve root compression. Additionally, this superior facet joint also participates in the construction of the lateral spinal canal, and thus it is also the main reason for lateral spinal stenosis [7, 8]. In the clinic, it has also been suggested that the lateral spinal stenosis and nerve root compression was rarely caused by the osteophytes of the inferior facet joint. Therefore, the inferior facet joint is not regarded as the decompression target and gains a great possibility to release the compressed nerve root without the resection of inferior facet joints. Our preliminary paper has proved the feasibility of that surgery and developed a new lumbar fusion technique called ELIF [9–11].

In the extraforaminal lumbar interbody fusion (ELIF) technique, only the superior facet joint is resected and the inferior facet joint and soft tissues attached behind it are retained. Compared with conventional transforaminal lumbar interbody fusion (TLIF), ELIF technique retains the posterior structures more completely and could potentially improve the immediate stability. Therefore, we analyzed the lumbar biomechanical stability of ELIF surgery by using 12 cadaveric spine specimens [12]. To make this analysis of the biomechanical stability more accurate, the 3D finite-element method was employed. In this study, L3–L5 ELIF and TLIF 3D finite-element models with different internal fixation and fusion methods were established. The model stability and stress of the pedicle screws, connecting rods and interbody fusion cages were tested under different operating conditions, namely, anterior flexion, posterior extension, left and right lateral flexion, and left and right rotation.

## 2. Data and Methods

### 2.1. Ethics Statement

This study has been reviewed and approved by the ethics committee of Shanghai East Hospital, Tongji University School of Medicine.

### 2.2. Establishment of 3D Finite-Element Model

**Figure 1:**
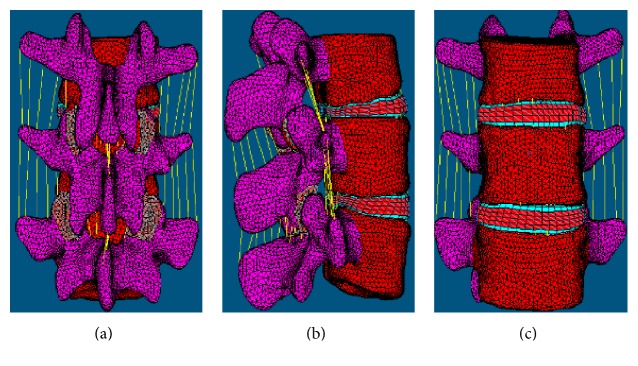
The intact model. (a) Posterior view. (b) Lateral view. (c) Anterior view.

In September 15, 2015, a 26-year-old man (height, 172 cm; weight, 67 kg; body mass index, 22.6 kg/m2) diagnosed with L4-L5 lumbar disc herniation was recruited for a preoperative lumbar computed tomography scan from T12 level to pelvic inlet using a dual source scanner (SOMATOM Definition Flash; Siemens Medical Solutions Inc., Forchheim, Germany). This participant provided the written informed consent to participate in this study. The scanning parameters were as follows: tube current = 250 mA, tube voltage = 120 kV, scanning slice thickness = 1.0 mm, and reconstruction slice thickness = 1.0 mm. The data in the commonly used DICOM 3.0 format were read using the medical finite-element modeling software Simpleware 2.0 (Simpleware Ltd., Exeter, UK), and an L3–L5 3D geometric model was established. We utilized the finite-element preprocessing software HyperMesh (Altair Engineering, Troy, USA) and select the appropriate element types and materials for mesh generation. The mesh model includes 124,528 elements and the mesh size is 2 mm. The materials for the various parts of the input model and their characteristics including elastic modulus and Poisson ratio are listed in Table 1 [13–17]. An intact L3–L5 segment finite-element model was shown in Figure 1.

### 2.3. Establishment of Models with Different Fusion and Internal Fixation Methods

**Figure 2:**
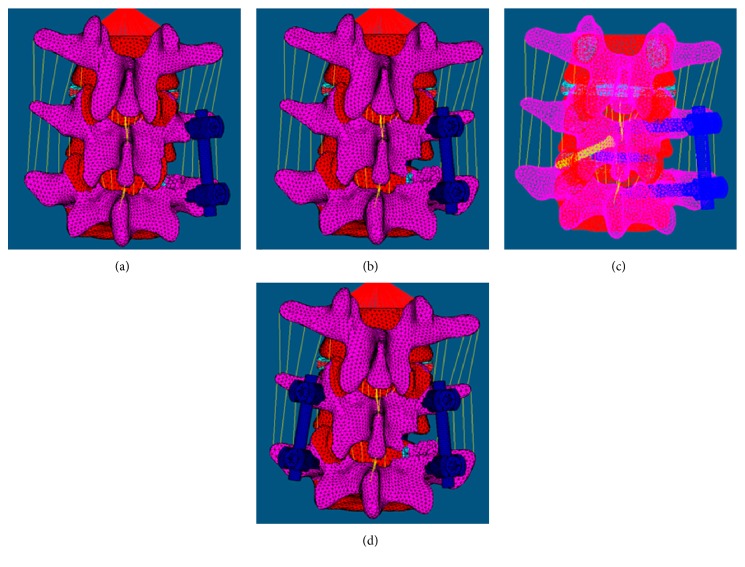
Finite-element model of ELIF and TLIF under different internal fixation modes. (a) ELIF + UPS. (b) TLIF + UPS. (c) ELIF + UPS + TLFS. (d) TLIF + BPS. ELIF: extraforaminal lumbar interbody fusion; TLIF: transforaminal lumbar interbody fusion; UPS: unilateral pedicle screw fixation; BPS: bilateral pedicle screw fixation; TLFS: translaminar facet screw.

Images of the pedicle screw system and intervertebral fusion cage (DePuy Spine, Johnson & Johnson, New Jersey, USA) in the IGES format were imported into HyperMesh, and the finite-element model was constructed for ELIF and TLIF based on the requirements listed below. The elastic modulus for screws in ELIF and TLIF groups was 110,000 MPa and the Poisson ratio was 0.3. The intervertebral fusion cage was a bullet-type cage with dimensions of 9 mm × 11 mm × 27 mm, and the elastic modulus and Poisson ratio were 3700 MPa and 0.25, respectively. The screw diameter was 6.0 mm, and length was 45 mm. The implanting angle of fusion cage was 80° at the spine sagittal plane in the ELIF group and 45° in the TLIF group. In both groups, the fusion cage was placed into the intervertebral space obliquely from the right side. The experimental groups were divided into as follows: ELIF with unilateral pedicle screw (ELIF + UPS), TLIF with unilateral pedicle screw (TLIF + UPS), TLIF with bilateral pedicle screws (TLIF + BPS), and ELIF with unilateral pedicle screw + translaminar facet screw (ELIF + UPS + TLFS). The designs of the experimental models were exactly based on the clinical surgical approaches. In the ELIF + UPS group, the superior facet joint of the L5 vertebra was removed along with the entire nucleus pulposus in the L4-L5 disc and the right posterior two-thirds of the fibrous ring. The posterior supraspinous ligament, interspinous ligament, spinous process, and the left side structures were retained. The pedicle screws were placed in the L4 and L5 pedicles on the right side (the entry point of each screw was the transition point of the superior facet joint and transverse process, and the screw was inserted at a 45° angle with the sagittal plane; Figure 2). In the TLIF + UPS group, the inferior facet joint of the L4 vertebra and the superior facet joint of the L5 vertebra were removed. The entire nucleus pulposus in the L4-L5 disc and the right posterior two-thirds of the fibrous ring were resected. The posterior supraspinous ligament, interspinous ligament, spinous process, and left structures were retained. The pedicle screws were placed in the right pedicles of the L4 and L5 vertebrae (the entry point of each screw was the traditional entry point, and the screws were inserted at a 15° angle with the sagittal plane; Figure 2). In the TLIF + BPS group, the decompression range was the same as that in the TLIF + UPS group. The pedicle screws need to be further implanted at the left slide pedicles compared to the TLIF + UPS group. The decompression range in the ELIF + UPS + TLFS group was the same as that in the ELIF + UPS group. Once pedicle screws were placed in the right L4 and L5 pedicles, a screw was implanted in the contralateral facet joint through the lamina (Figure 2).

### 2.4. Loading and Recording Methods

The inferior surface of the L5 vertebral body was totally fixed. Surface loading was applied on the superior surface of the L3 vertebral body, vertically in the downward direction and with uniform distribution on the entire superior endplate of the L3 vertebral body. The load applied on the model was 400 N and the additional force from motion was 6 N·m [18]. The data were input in Abaqus 6.10 (Dassault Systemes Simulia Corp., Providence, RI, USA), and calculations were performed under six operating conditions: lumbar spine anterior flexion, posterior extension, left and right lateral flexion, and left and right rotation. The main parameters observed were as follows: (1) L4-L5 range of motion (ROM), represented by the segmental angular displacement. The spatial coordinates of 4 points (the most forward point, most backward point, most leftward point, and most rightward point) on the superior surfaces of the L4 and L5 vertebrae were measured and connected with lines. The angles between the lines represented the angles between the superior surfaces of two neighboring vertebral bodies. The absolute value of the difference in these angles before and after loading was the angular displacement of the L4-L5 segments. (2) Stress diagrams were used to represent the stress on the pedicle screws, connecting rods and intervertebral fusion cages, under six operating conditions.

### 2.5. Validation Process of Finite-Element Model

This intact L3-L5 finite-element model was validated by comparing the intact finite-element model reported by the literature [17–19].

## 3. Results

### 3.1. Verification of the Effectiveness of the Models

**Figure 3:**
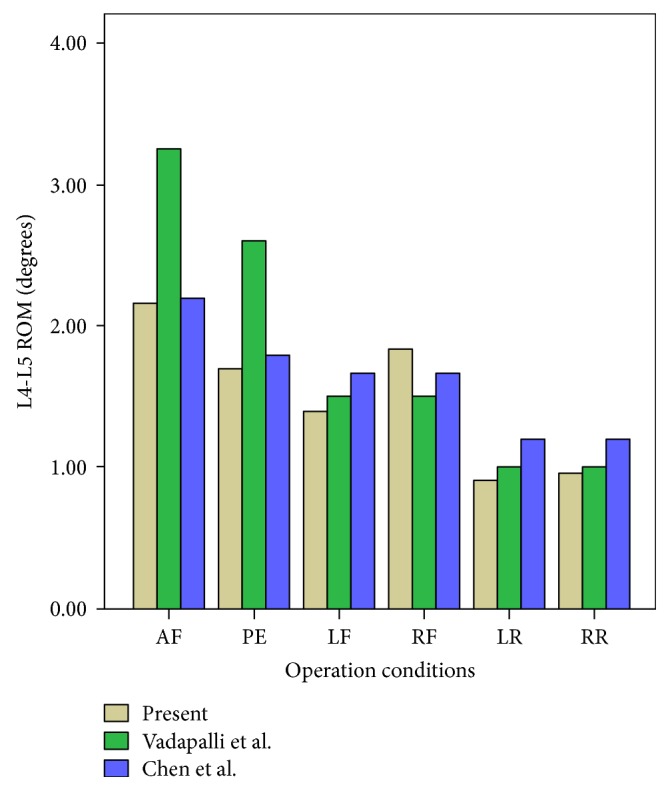
Comparison between the current intact model and previous studies for the validation. Comparison with Vadapalli et al. and Chen et al. AF: anterior flexion; PE: posterior extension; LF: lateral flexion; RF: right flexion; LR: left rotation; RR: right rotation.

The whole L3–L5 3D nonlinear finite-element model consists of the cortical bone shell, endplate, cancellous bone core, intervertebral disc (ground substance, collagen fibers, and nucleus pulposus), and 7 types of ligaments, yielding a total of 13 types of materials (Table 1). The model contained 124,528 units and 49,235 nodes. After defining the constraints and loading conditions for this model, the angular displacements of the L4-L5 segments in intact model were calculated under six operating conditions (lumbar spine anterior flexion, posterior extension, left and right lateral flexion, and left and right rotation). The results were basically consistent with those of the finite-element study by Chen et al. (Figure 3). Thus, we concluded that this 3D finite model was effective under certain conditions and could be applied for clinical and experimental studies [17–19].

### 3.2. L4-L5 ROM

**Figure 4:**
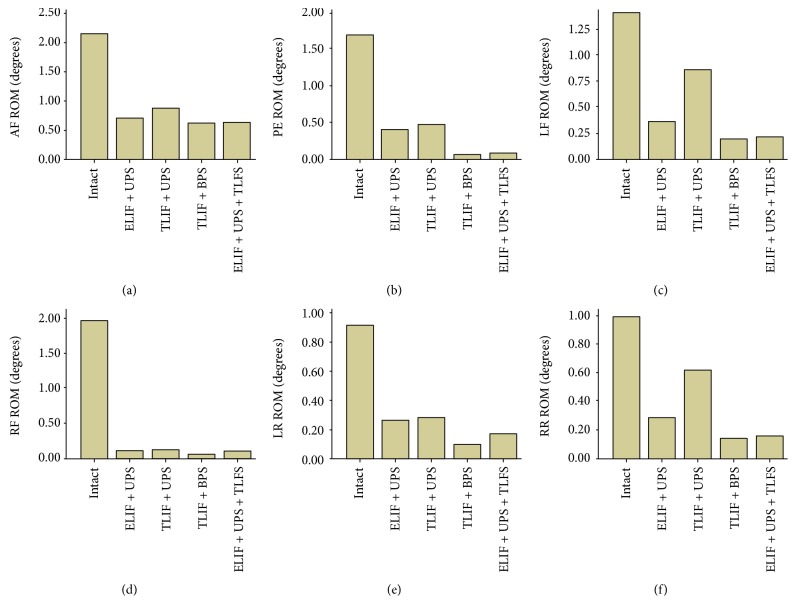
Range of motion at L4-L5 in intact and fixation models under six operation conditions. The ROM was smaller in all internal fixation groups than in the intact group. Furthermore, the ROM was smaller in the ELIF + UPS group than in the TLIF + UPS group, under all operating conditions, especially left lateral flexion and right rotation. The ROM was smaller in the TLIF + BPS group than in the ELIF + UPS group. Similarly, in the ELIF + UPS + TLFS group, the ROMs were much smaller than those in the ELIF + UPS group. When compared with the TLIF + BPS group, the ROM in the ELIF + UPS + TLFS group shows no obvious difference in the range of experimental loading. AF: anterior flexion; PE: posterior extension; LF: left lateral flexion; RF: right lateral flexion; LR: left rotation; RR: right rotation.

The ROMs were smaller in all internal fixation groups than those in the intact group (Table 2, Figure 4). Furthermore, the ROMs were smaller in the ELIF + UPS group than those in the TLIF + UPS group under all operating conditions, especially left lateral flexion and right rotation. During left lateral flexion and right rotation, the ROMs of ELIF + UPS were reduced by 56.32% and 53.33%, respectively. The ROMs were smaller in the TLIF + BPS group than those in the ELIF + UPS group, and the percentages of decrease were as follows: anterior flexion 11.27%, posterior extension 80.49%, left lateral flexion 42.11%, right lateral flexion 45.45%, left rotation 61.54%, and right rotation 50.00% (Table 2 and Figure 4). Similarly, in the ELIF + UPS + TLFS group, the ROMs were much smaller than those in the ELIF + UPS group. The percentages of decrease were as follows: anterior flexion 9.86%, posterior extension 75.61%, left lateral flexion 36.84%, right lateral flexion 9.09%, left rotation 34.62%, and right rotation 42.86% (Table 2, Figure 4). When compared to those in the TLIF + BPS group, the ROMs in the ELIF + UPS + TLFS group show no significant difference in the range of experimental loading.

### 3.3. Stress Analysis of the Pedicles, Connecting Rods, and Intervertebral Fusion Cages in the ELIF and TLIF Finite-Element Models

**Figure 5:**
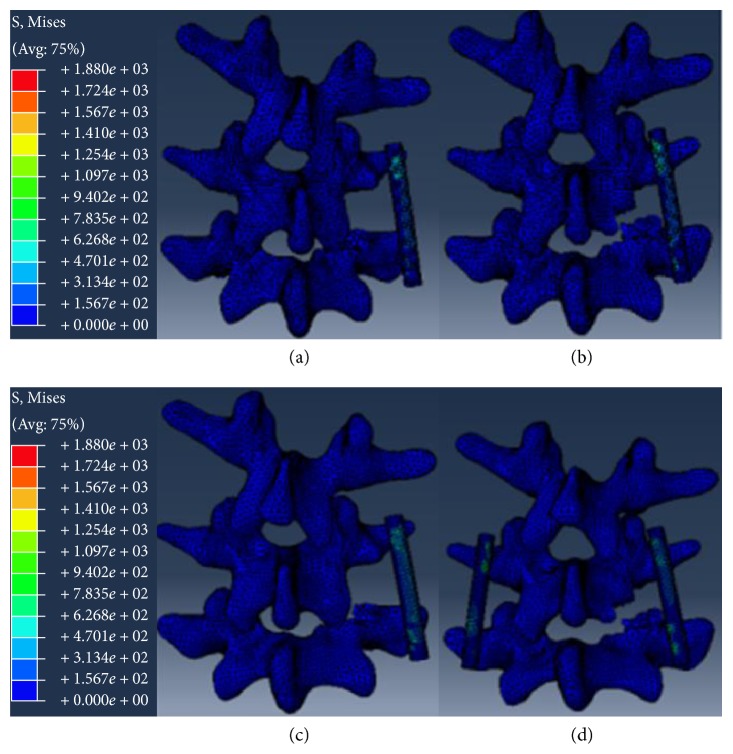
Stress analysis of the connecting rods in the ELIF and TLIF finite-element models under right lateral flexion. (a) ELIF + UPS. (b) TLIF + UPS. (c) ELIF + UPS + TLFS. (d) TLIF + BPS. The maximum stress concentration point on the connecting rod was the junction between the screw and its head in the ELIF and TLIF models. The stress on the proximal end was greater than that on the distal end. The stresses on the connecting rod under all operating conditions were greater in the case of unilateral pedicle screw fixation than in the case of bilateral pedicle screw fixation. Furthermore, the stress on the connecting rod was smaller in the ELIF + UPS group than in the TLIF + UPS group, especially under right lateral flexion. With additional contralateral translaminar facet screw fixation, the stress on the connecting rod was decreased as compared with simple unilateral pedicle screw fixation.

The maximum stress concentration point on the connecting rod was the junction between the screw and its head in the ELIF and TLIF models. The stress on the proximal end was greater than that on the distal end. The stresses on the connecting rod under all operating conditions were greater in the case of unilateral pedicle screw fixation than those in the case of bilateral pedicle screw fixation. Furthermore, the stresses on the connecting rods were smaller in the ELIF + UPS group than those in the TLIF + UPS group, especially under right lateral flexion (Figure 5). With additional contralateral translaminar facet screw fixation, the stresses on the connecting rods were decreased as compared to only unilateral pedicle screw fixation. The stresses on the fusion cages did not significantly differ between the ELIF and TLIF groups under six operating conditions (Figure 5).

## 4. Discussion

TLIF has gained increasing popularity in the treatment of degenerative lumbar disease due to its advantages including direct decompression on the nerve root and less risk of complications and good fusion result [20–22]. However, TLIF as the classical posterior lumbar interbody fusion technique can also lead to lumbar muscle injury and reduce the postoperation lumbar spine stability [23, 24]. To improve the postoperation lumbar spine stability, a large number of new fusion techniques such as ALIF, DLIF, and OLIF have been designed and applied [25–28]. Although the stability has been enhanced with these techniques, the outcome of decompression on nerve root cannot be ensured because direct decompression on nerve root cannot be accessed through these surgical approaches. Therefore, we designed and tested the biomechanical stability of the new fusion technique (ELIF) based on the direct decompression and less trauma principles. In this study, the finite-element analysis of this fusion technique under different internal fixation conditions was performed to further evaluate the lumbar spine stability of this fusion surgery.

Finite-element models are widely applied in understanding of biomechanical function of the spine due to the effective stimulation, high repeatability, and less cost [29]. Before a new surgery technique is used in the clinic, the corresponding 3D model can be designed to test the reliability and safety of this new technique. The analyses indicated that lumbar stability was better in the case of ELIF with unilateral pedicle screw fixation than in the case of TLIF with unilateral pedicle screw fixation, under six operating conditions, especially, left lateral flexion and right rotation. The reason for the above finding is that ELIF with unilateral pedicle screw fixation can retain part of the superior facet joint to associate with the inferior facet joint to limit left lateral flexion and right rotation of the lumbar spine. Additionally, the retention of posterior capsular ligaments in this joint can also be the beneficial factor to restrict lumbar movement. Last but not least, pedicle screws in ELIF with unilateral fixation group had greater extraversion, which was also beneficial to improve stability. Therefore, ELIF with unilateral fixation gained a better lumbar stability. However, the stability of the above ELIF was significantly weaker than that of TLIF with bilateral pedicle screw fixation. It means that although ELIF with unilateral pedicle screw fixation improved stability compared by TLIF with unilateral pedicle screw fixation, it still could not reach the stability achieved by TLIF with bilateral pedicle screw fixation. Moreover, the stability of ELIF with unilateral pedicle screw fixation could be improved by additional contralateral translaminar facet screw fixation. Thus, ELIF with unilateral pedicle screw fixation supplemented with translaminar facet screw fixation could meet the stability requirements for lumbar fusion surgeries [30, 31]. Stress analysis of the pedicle screws, connecting rods, and intervertebral fusion cages have also been compared between the ELIF and TLIF finite-element models. The stress on the connecting rod was smaller in the ELIF + UPS group than that in the TLIF + UPS group. It is because that part of L5 superior facet joint in the ELIF + UPS group was retained so that it can be associated with L4 inferior facet joint. Further, this joint can partly share the stress on the connecting rod. Additionally, the retention of posterior capsular ligaments in this joint can also provide the beneficial factor to share the stress on the connecting rod. Therefore, the possibility of rod breakage would theoretically be lower in the ELIF + UPS group than in TLIF + UPS group. As the stress on fusion cages is concerned, there was no significant difference between ELIF groups and TLIF groups. It is because the function of fusion cage was to support the anterior pillar, and thus the retention of the posterior pillar structures had little impact on the stress exerted on the fusion cage. Additionally, ELIF can achieve the direct decompression on nerve root and decrease internal fixation cost which consists of the majority of the whole cost in china. Therefore, this new ELIF surgery can potentially be a stable fusion technique which use a more invasive and economic internal fixation than classical TLIF with bilateral pedicle screw fixation.

Although this study has achieved primary success, more work needs to be done. Firstly, material properties in these 3D models were simplified and idealized. Although these simplifications were reasonable, it influenced the exact ROM value. Thus, the results obtained in these analyses reflect the difference of ROMs among groups instead of the exact ROM value. Secondly, the soft tissue environment was different between these models and human lumbar spines because the whole spinal model including lumbar spine muscle failed to be established.

## Figures and Tables

**Table 1:** Material property of spinal components and implants.

Component	Young's modulus (MPa)	Poisson's ratio	Cross section (mm^2^)
Cortical bone	12000.0	0.30	
Endplate	1200.0	0.29	
Cancellous bone	100.0	0.30	
Annulus ground substance	4.2	0.45	
Nucleus pulposus	1.0	0.49	
Annulus fiber	450.0	0.45	
Anterior longitudinal ligaments	20.0	0.30	63.7
Posterior longitudinal ligaments	20.0	0.30	20.0
Intertransverse ligament	58.7	0.30	3.6
Ligamentum flavum	19.5	0.30	40.0
Interspinous ligament	11.6	0.30	40.0
Supraspinous ligament	15.0	0.30	30.0
Capsular ligament	32.9	0.30	60.0
Pedicle screws and rod	110000.0	0.28	20.0
PEEK cage	3600.0	0.25	

**Table 2:** The L4-L5 range of motion (ROM) in different groups under six operating conditions.

ROM (*n* = 12)	AF (°)	PE (°)	LF (°)	RF (°)	LR (°)	RR (°)
Control	2.16	1.70	1.40	1.84	0.90	0.96
ELIF + UPS	0.71	0.41	0.38	0.11	0.26	0.28
TLIF + UPS	0.88	0.48	0.87	0.12	0.28	0.60
TLIF + BPS	0.63	0.08	0.22	0.06	0.10	0.14
ELIF + UPS + TLFS	0.64	0.10	0.24	0.10	0.17	0.16
Percentage decrease from control to ELIF + UPS	67.13%	75.88%	72.86%	94.02%	68.89%	70.83%
Percentage decrease from control to TLIF + UPS	59.26%	71.76%	37.86%	93.48%	52.45%	37.50%
Percentage decrease from TLIF + UPS to ELIF + UPS	19.32%	14.58%	56.32%	8.33%	7.14%	53.33%
Percentage decrease from ELIF + UPS to TLIF + BPS	11.27%	80.49%	42.11%	45.45%	61.54%	50.00%
Percentage decrease from ELIF + UPS to ELIF + UPS + TLFS	9.86%	75.61%	36.84%	9.09%	34.62%	42.86%
Percentage decrease from ELIF + UPS + TLFS to TLIF + BPS	1.56%	20.00%	8.33%	40.00%	41.18%	12.50%

ELIF: extraforaminal lumbar interbody fusion; UPS: unilateral pedicle screw; TLIF: transforaminal lumbar interbody fusion; BPS: bilateral pedicle screw; TLFS: translaminar facet screw; AF: anterior flexion; PE: posterior extension; LF: lateral flexion; RF: right flexion; LR: left rotation; RR: right rotation.
